# Novel Role for Animal Innate Immune Molecules: Enterotoxic Activity of a Snail Egg MACPF-Toxin

**DOI:** 10.3389/fimmu.2020.00428

**Published:** 2020-03-13

**Authors:** Matías L. Giglio, Santiago Ituarte, Andrés E. Ibañez, Marcos S. Dreon, Eduardo Prieto, Patricia E. Fernández, Horacio Heras

**Affiliations:** ^1^Instituto de Investigaciones Bioquímicas de La Plata “Prof. Dr. Rodolfo R. Brenner” (INIBIOLP), CONICET, CCT-La Plata, Universidad Nacional de la Plata (UNLP), La Plata, Argentina; ^2^División de Vertebrados, Facultad de Ciencias Naturales y Museo (FCNyM), Universidad Nacional de La Plata, La Plata, Argentina; ^3^Cátedra de Bioquímica y Biología Molecular, Facultad de Ciencias Médicas, Universidad Nacional de la Plata (UNLP), La Plata, Argentina; ^4^Instituto de Investigaciones Físico-químicas Teóricas y Aplicadas (INIFTA), CONICET, CCT-La Plata, Universidad Nacional de La Plata, La Plata, Argentina; ^5^Facultad de Ciencias Veterinarias (FEV), Instituto de Patología B. Epstein, Cátedra de Patología General Veterinaria, Universidad Nacional de La Plata (UNLP), La Plata, Argentina

**Keywords:** *Pomacea maculata*, snail reproduction, PV2, intestinal morphology, antipredator defense, AB toxin, cytotoxicity

## Abstract

Gastropod Molluscs rely exclusively on the innate immune system to protect from pathogens, defending their embryos through maternally transferred effectors. In this regard, *Pomacea* snail eggs, in addition to immune defenses, have evolved the perivitellin-2 or PV2 combining two immune proteins into a neurotoxin: a lectin and a pore-forming protein from the Membrane Attack Complex/Perforin (MACPF) family. This binary structure resembles AB-toxins, a group of toxins otherwise restricted to bacteria and plants. Many of these are enterotoxins, leading us to explore this activity in PV2. Enterotoxins found in bacteria and plants act mainly as pore-forming toxins and toxic lectins, respectively. In animals, although both pore-forming proteins and lectins are ubiquitous, no enterotoxins have been reported. Considering that *Pomacea* snail eggs ingestion induce morpho-physiological changes in the intestinal mucosa of rodents and is cytotoxic to intestinal cells in culture, we seek for the factor causing these effects and identified PmPV2 from *Pomacea maculata* eggs. We characterized the enterotoxic activity of PmPV2 through *in vitro* and *in vivo* assays. We determined that it withstands the gastrointestinal environment and resisted a wide pH range and enzymatic proteolysis. After binding to Caco-2 cells it promoted changes in surface morphology and an increase in membrane roughness. It was also cytotoxic to both epithelial and immune cells from the digestive system of mammals. It induced enterocyte death by a lytic mechanism and disrupted enterocyte monolayers in a dose-dependent manner. Further, after oral administration to mice PmPV2 attached to enterocytes and induced large dose-dependent morphological changes on their small intestine mucosa, reducing the absorptive surface. Additionally, PmPV2 was detected in the Peyer's patches where it activated lymphoid follicles and triggered apoptosis. We also provide evidence that the toxin can traverse the intestinal barrier and induce oral adaptive immunity with evidence of circulating antibody response. As a whole, these results indicate that PmPV2 is a true enterotoxin, a role that has never been reported to lectins or perforin in animals. This extends by convergent evolution the presence of plant- and bacteria-like enterotoxins to animals, thus expanding the diversity of functions of MACPF proteins in nature.

## Introduction

The innate immune system is a protective line of defense toward foreign organisms present, to some extent, in all multicellular organisms. Mollusks, like all other invertebrates, rely exclusively on the innate immune system, using both cellular and humoral defense lines (1). Among the humoral effectors are reactive oxygen species, lectins, antimicrobial peptides, proteases, protease inhibitors, and pore-forming proteins, many of which are awaiting functional characterizations (2). In addition to protecting the adults, it has been shown that several of these compounds are also maternally transferred to eggs where they would provide immunity to the developing embryos. For instance, recent proteomic studies revealed the presence of many proteins with defensive roles in the egg fluid—referred to as perivitelline fluid (PVF)—of several snail species including *Biomphalaria glabrata, Marisa cornuarietis, Pomacea diffusa, P. canaliculata*, and *P. maculata* (3–7). Among these proteins, called perivitellins, an evolutionary novelty arose in the eggs of some *Pomacea* species, in which two immune effectors, a perforin from the Membrane Attack Complex and Perforin (MACPF) family and a tachylectin, combined and formed a neurotoxin, the perivitellin-2 or PV2 (8, 9). This binary structure is unique among animals and resembles those of bacterial and plant AB toxins, where a ¨B¨-moiety acts as a delivery unit of a toxic ¨A¨-moiety (10, 11). Unlike AB toxins from bacteria or plants, snail PV2 contains a unique arrangement of two AB toxins in a head-to-tail fashion (12). Interestingly, many of these AB toxins, such as the cholera toxin (CT), heat labile toxin (LT), and shiga toxins (Stxs) from bacteria and the type-2 ribosome inactivating proteins (RIPs) from plants, act as enterotoxins (11), an unexplored function in PV2.

Enterotoxins are a group of toxic proteins that target the digestive system. In many bacteria they intervene in pathogenic processes (13, 14) and most of them are cytotoxic to intestinal cells usually by forming pores in the plasma membrane hence known as pore-forming toxins (PFTs) (13, 15, 16). On the other hand, plant enterotoxins are mostly toxic lectins, particularly abundant in seeds, that play a role in the defense against herbivory (17–19). Both bacteria and plant enterotoxins adversely affect gut physiology and/or morphology usually by cytotoxicity on intestinal cells, disruption of the brush border, and changes in the digestive, absorptive, protective or secretory functions, that could eventually lead to death (14, 17, 19). Moreover, some bacterial enterotoxins elicit inflammatory processes and immune system activation in mammals (14, 15).

Remarkably, no enterotoxins have been reported in animals, although both pore-forming proteins and lectins are widely distributed (20, 21). Even more, when these animal proteins act as toxins they always target other systems (8, 9, 21, 22). This lack of enterotoxins is surprising given that plant and animal embryos are often exposed to similar selective pressures by predators and pathogens alike. However, recent studies in *Pomacea* snails have reported egg defensive compounds targeting the digestive system suggesting the presence of enterotoxins. For instance, ingestion of *P. canaliculata* PVF decreases rat growth rate, induces morphological changes in the small intestine mucosa, and decreases the absorptive surface in mice and rats (9, 23, 24). This PVF also showed cytotoxic effects on intestinal cells of the Caco-2 line (23). Moreover, the gastrointestinal tract of mice exposed to *P. canaliculata* PVF increases the permeability of the digestive barrier (24). Although the compounds responsible of these enterotoxic effects were unknown, some perivitellins with non-toxic defensive properties targeting the digestive system were isolated from *Pomacea* eggs such as protease inhibitors and non-digestible storage proteins (24–28). However, as PV2 is a toxin with the same structural domains as plant and bacteria enterotoxins, we wondered if it would be responsible for the enterotoxic effects observed for the *Pomacea* PVF.

Thus, the aim of this work was to evaluate the enterotoxic capacity of PmPV2. Using *in vitro* and *in vivo* approaches, we evaluated the ability of PmPV2 to resist enzymatic proteolysis and extreme pH, as well as its cytotoxicity, capacity to bind to intestinal cells, disrupt cell monolayers, cause morphological changes and traverse the intestinal barrier inducing adaptive immunity *via oral*. We found that PmPV2 was able to withstand a wide range of pHs and gastrointestinal proteases both *in vitro* and *in vivo*. Then, it binds to enterocytes which is followed by necrosis. Mice fed with the toxin showed strong morphological changes in their small intestine and a reduction of the absorptive surface. Additionally, PmPV2 was detected in the Peyer's patches where it induced cell apoptosis. PmPV2 triggered oral immunization indicating it can reach the circulatory system. All these results provide the first evidence that PmPV2, an animal PFT, besides neurotoxicity, exerts enterotoxicity when ingested further potentiating the multiple defenses of *Pomacea* eggs.

## Results

### Stability at Physiologically Relevant PHs and Gastrointestinal Environments

**Figure 1:**
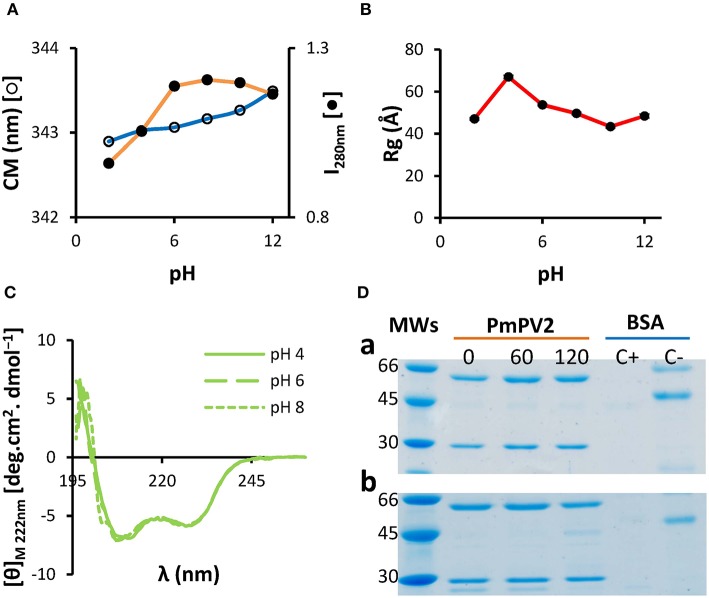
PmPV2 is structurally stable in a wide range of pH and resists *in vitro* gastrointestinal digestion. **(A–C)** PmPV2 stability at different pH values. Stability was measured following the changes in: **(A)** Trp environment from pH 2.0 to pH 12.0, depicted as center of mass (CM) and fluorescence intensity at 280 nm (I_280_); **(B)** gyration radii (*Rg*) obtained by SAXS; **(C)** secondary structure by CD spectra in the far-UV region at pH 4.0 (solid line), pH 6.0 (dashed line) and pH 8.0 (dotted line). **(D)** Gastric phase (a). PmPV2 exposed for 0, 60 and 120 min to pepsin at pH 2.5. MWs: molecular weight standard (kDa); Duodenal phase (b). PmPV2 exposed for 0, 60, and 120 min to trypsin at pH 8.5 after 120 min of gastric phase. Positive control (C+): BSA with enzyme, negative control (C–): BSA without enzyme.

A selective pressure faced by a defensive egg protein when ingested by a predator is the gastrointestinal tract pH and proteases. Therefore, stability of PmPV2 in a wide range of pH was analyzed by fluorescence, small angle X-ray scattering (SAXS) and circular dichroism (CD) spectroscopies. Fluorescence spectra did not change significantly between pH 4.0–10.0, while a change in emission spectra at extreme pH values, 2.0 and 12.0, was observed (Figure 1A). Likewise, SAXS analysis showed no significant changes in gyration radius (*Rg*) between pH 6.0 and 10.0 and protein denaturation was only evident at extreme pH values (Figure 1B). However, *Rg* increased from 53.7 nm (at pH 6.0) to 67.0 nm (at pH 4.0), together with its maximum intramolecular distance (*Dmax*), which went from 108 to 134 nm, at pH 6.0 and 4.0, respectively (Figure S1); molecular mass only increased slightly (Figure S1), indicating the protein expanded without oligomerization or aggregation. Far-UV CD spectra region showed structural stability in the pH range 4.0–8.0 (Figure 1C). These results indicate remarkable structural stability of the protein in a wide range of pH values and an expansion event at pH 4.0.

In addition, the susceptibility of PmPV2 to protease activity *in silico* and *in vitro* was also evaluated. *In silico* digestion showed that both PmPV2 subunits have putative cleavage sites for both pepsin and trypsin, indicating it is potentially susceptible to proteases. In particular, pepsin has 48 and 23 cleavage sites for the heavy (PmPV2-67) and light subunit (PmPV-31), respectively, while trypsin has 151 cleavage sites in PmPV2-67 and 76 in PmPV2-31. However, *in vitro* digestion assay showed that PmPV2 was able to withstand both gastric and duodenal phases with only minor protein degradation in the latter (Figure 1D). This result agreed with the immunodetection of PmPV2 attached to intestinal mucosae after oral administration to mice (*see below*).

### Binding to Intestinal Cells and Effects on Cell Morphology and Small Intestinal Mucosa

**Figure 2:**
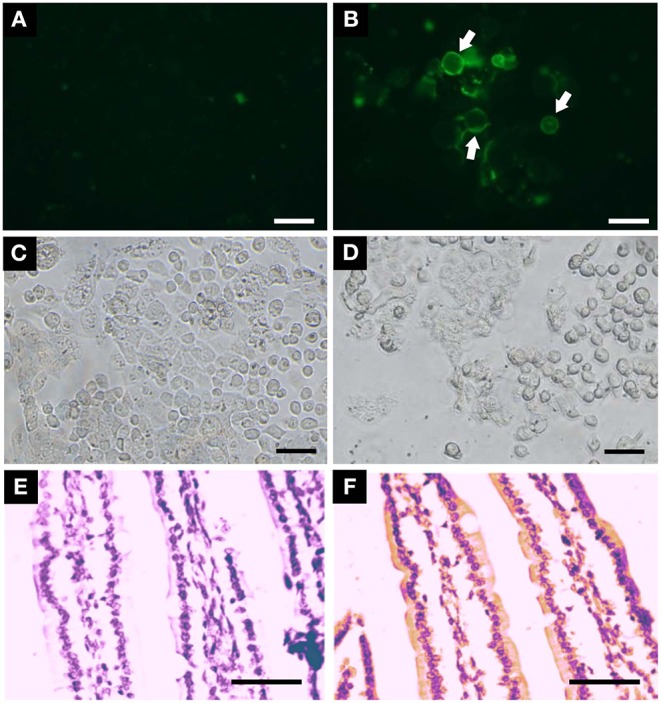
PmPV2 binds to intestinal cells. **(A,B)** PmPV2 binding to intestinal cells in culture. Caco-2 cells were incubated for 1 h with Alexa-488 labeled BSA as control **(A)** or with PmPV2 **(B)**. PmPV2 attached to cell surface is highlighted with arrowheads. Bar 25 μm. **(C,D)** Same sections of BSA **(C)** and PmPV2 **(D)** treatments shown in A and B under bright field. Bar 25 μm. **(E,F)** Immunolocalization of PmPV2 at brush border intestinal mucosae of mice fed on a diet without **(E)** and with **(F)** 400 μg PmPV2 (PmPV2 location appears as yellowish regions). Bar 50 μm.

Toxins that enter the predator's body by ingestion have first to bind to epithelial cells to be internalized to reach its target or exert its functions. In this regard, we analyzed PmPV2 binding to Caco-2 cells and enterocytes from the small intestine mucosa. Whereas, Caco-2 cells treated with Alexa-BSA showed no label (Figure 2A), after the incubation with Alexa-labeled PmPV2 the surface of some of these cells was marked, indicating that the toxin attaches to the cell membrane, particularly to round-shaped and partially detached cells (Figure 2B). These morphological changes were presumably caused by the toxin since the cells that remained attached to the surface showed mild or no labeling. Accordingly, most Caco-2 cells treated with BSA remained attached and conserved a flat shape (Figure 2C) whereas PmPV2-treated cells showed an increased level of detached, rounded-shape cells (Figure 2D). In the same way, PmPV2 was immunodetected bound to the enterocyte surface of small intestine (Figures 2E,F), indicating that the toxin withstands gastrointestinal digestion *in vivo* and reaches the small intestine in an active form.

**Figure 3:**
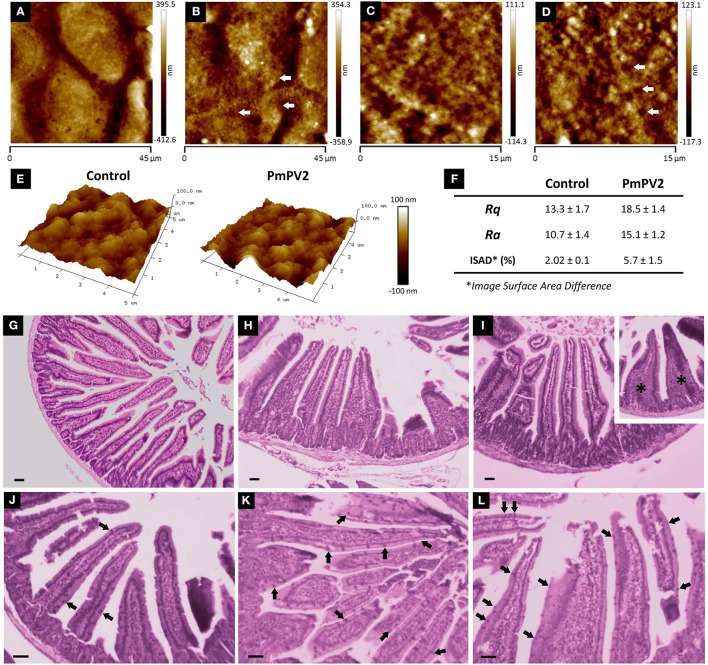
PmPV2 affects Caco-2 and small intestine morphology. **(A–D)** AFM images obtained in tapping topography. Gross morphology of control cells **(A,C)** and PmPV2-treated Caco-2 cells **(B,D)** (**A,B**: 45 × 45 μm^2^, **C,D**: 15 × 15 μm^2^). Ring-like structures are highlighted with arrows. **(E)** 3D AFM topography images of control and PmPV2-treated Caco-2 cells at 10 x 10 μm^2^ showing cell surface details. **(F)** *Ra, Rg* and *Image Surface Area Difference* values for control and PmPV2-treated Caco-2 cells. **(G–I)** Intestinal sections stained with HE of control mice **(G)** and mice treated with one **(H)** or four **(I)** doses of 400 μg of PmPV2. *Inset*: tongue-like fused villi (asterisks). **(J–L)** Intestinal sections stained with PAS. Globet cells are positively stained (some highlighted with arrowheads) in both control mice **(J)** and mice treated with one **(K)** and four **(L)** doses of 400 μg of PmPV2 each. Bars 100 μm.

After having determined that PmPV2 binds to intestinal cell surfaces, we evaluated its effects on cell morphology and small intestinal mucosa. The alterations of Caco-2 cells were evaluated by quantifying changes on their surface with AFM. Whereas control cells were oval-shaped, with a maximum diameter of ~35 μm and well-defined cell limits (Figure 3A), cells exposed to PmPV2 showed irregular form, granulated aspect and diffuse cell limits (Figure 3B). Additionally, small rounded structures of ~1.8 μm of an unknown nature were observed on treated cells. Under greater magnification cell membranes showed a more homogeneous surface in control cells (Figure 3C) than in treated cells (Figure 3D). An important increase in membrane roughness was observed in PmPV2-treated cells: Roughness of 25 μm^2^ sections was 40% higher in treated than in control cells as reflected in their *Rq* and *Ra* parameters (Figures 3E,F), while the ISAD increased ~200% in treated cells (Figure 2F).

In addition to cell-level alterations, oral administration of PmPV2 caused notable morphological changes in the small intestinal mucosae of mice (Figures 3G–L). In comparison with control animals (Figure 3G), treated mice showed shortening and widening of villi, which were already evident after one dose of PmPV2 (Figure 3H) and even more evident in animals receiving four doses (Figure 3I). In the latter group, fused “tongue-like” villi were also observed. Besides, duodenal mucosae of treated animals showed a higher number of goblet cells (Figures 3K–L) than that of the control group (Figure 3J).

We calculated the effect of PmPV2 on the absorptive surface using the parameter of Kisielinski et al. (29). Control groups have a mucosal-to-serosal amplification ratio (*M*) of 14.5 ± 1.5 (mean ± SD). Mice treated with PmPV2 showed a decrease of this ratio with a reduction of 10 and 12% in animals fed with one dose (*M* = 13.11 ± 1.66) and four doses (*M* = 12.82 ± 1.62), respectively (1 dose of PmPV2: *P* < 0.001; 4 doses of PmPV2: *P* < 0.0001, vs. control mice).

### Cytotoxicity and Cell Monolayer Disruption

**Figure 4:**
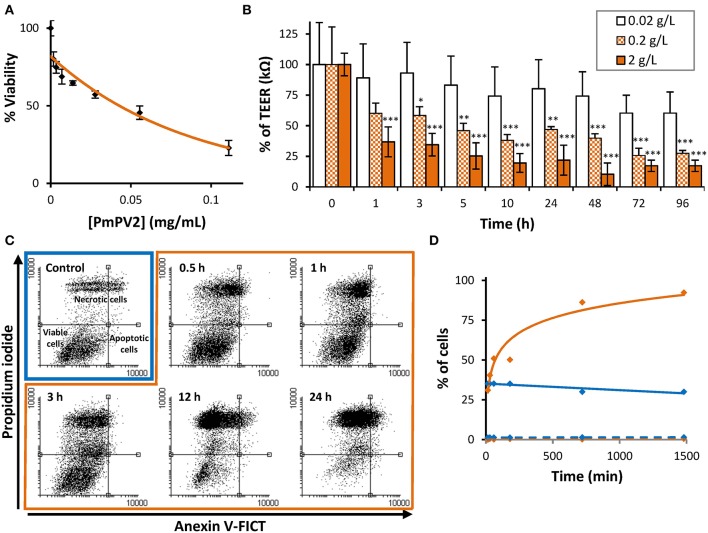
PmPV2 is toxic to Caco-2 cells and disrupts enterocyte monolayers. **(A)** Cytotoxic effect of PmPV2 on Caco-2 cells evaluated using MTT assay. **(B)** Capacity of PmPV2 to disrupt a enterocyte monolayers (TEER assay) at three concentrations. Results are expressed as Mean ± SEM of three replicates. *P<0.05, **P < 0.01, ***P <0.001. **(C)** Flow cytometry analysis showing type of cell death caused by PmPV2. Cells were doubly-labeled with anexin V-FICT/propidium iodide (PI), without (control, blue box) and with 0.05 mg/mL PmPV2 (orange box) at 0.5, 1, 3, 12 and 24 h. Viable cells: FICT^−^/PI^−^; Apoptotic cells: FICT^+^/PI^−^; Necrotic cells: FICT^−^/PI^+^ and FICT^+^/PI^+^. **(D)** percentage of apoptotic (dashed line) and necrotic (full line) cells obtained in **(C)**. Blue lines: control; orange lines: treated with PmPV2.

We tested the effect of PmPV2 on cultured intestinal absorptive cells and intestinal cell monolayers, which the toxin would encounter when ingested by a predator. For this, we tested Caco-2 cells, widely used as a model of the intestinal epithelia that we knew showed reduced viability when exposed to *P. canaliculata* PVF (23). These cells were much affected by the PmPV2 toxin in a dose-dependent manner, with 0.11 mg/mL PmPV2 inducing 80% of cell death after 24 h (Figure 4A). The effect of PmPV2 on Caco-2 cells was also analyzed measuring its capacity to alter differentiated cells in a highly attached monolayer by measuring the transepitelial electric resistance (TEER) at three toxin concentrations (Figure 4B). Cell monolayers exposed to PmPV2 showed a decrease in TEER in <1 h at the highest toxin concentration (2 g/L). At the intermediate concentration (0.2 g/L), the TEER showed a progressive decrease between 3 and 10 h; after 10 h the lower TEER value was sustained until the end of the experiment. The lowest toxin concentration (0.02 g/L) showed no effect on TEER within the duration of the experiment. Together these results pointed out that PmPV2 is responsible for the effect of snail eggs on digestive cells.

We, therefore, analyzed whether cell death was caused by lytic or non-lytic mechanisms (i.e., necrosis or apoptosis). To evaluate the toxic mechanism, cells with or without treatment with PmPV2 were analyzed by flow cytometry and changes in cell populations were quantified. The control group showed a basal level of apoptotic cells (<2%) and necrotic cells (around 35%), while most cells remained viable (Figures 4C,D). PmPV2 exposed group showed an increase in necrotic cells with the concomitant decrease in viable cells, while the apoptotic cell population showed no significant changes (Figures 4C,D). In PmPV2-treated cells, necrotic cell population showed two subpopulations of high and low propidium iodide labeling intensities (Figure 4C). Necrotic cells were evident after 30 min of exposure and 50% of lethality was reached in ~70 min (Figure 4D). In any case, apoptotic cells were always below 2%.

### Fate and Internalization in the Intestinal Mucosa

**Figure 5:**
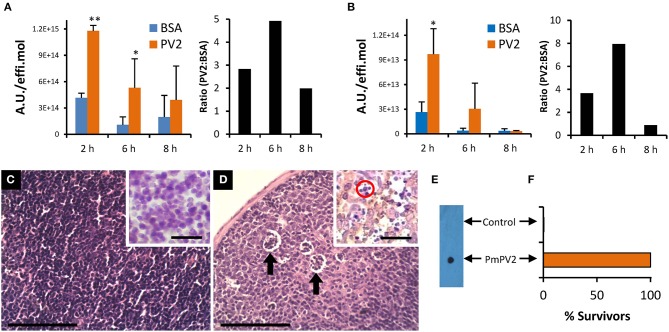
PmPV2 fate, internalization and immune response after oral ingestion in mice. **(A,B)** Fate and internalization of fluorescently-labeled PmPV2 in mice intestine **(A)** and Peyer's patches **(B)** at different times after ingestion. Results are expressed as the Mean ± SEM of A.U./effi.mol (*n* = 3) and are representative of two independent experiments. *Right panels*: PmPV2:BSA ratio of values from left panel. *P<0.05; **P<0.01. **(C,D)** Peyer's patches of control mouse **(C)** and PmPV2-treated mouse **(D)**. Cumuli of apoptotic cells are highlighted with arrows. Bar 100 μm. *Insets*: IHC using mouse IgG anti-Caspase 3 antibodies (1:200). Mice treated with PmPV2 showed positive reaction; Caspase-3 location appears as yellowish regions. Apoptotic bodies were also evident in treated mice (red circle). Bar 50 μm. **(E,F)** Mice oral immunization against PmPV2. **(E)** Dot blot analysis of sera from mice gavaged with PBS (Control) or with 800 μg PmPV2 (PmPV2). **(F)** Effect of lethal (1 mg) i.p. dosis of PmPV2 on control and immunized mice survival (*n* = 3). Please note graph does not include error bars because all of the three immunized animals survived and all of the three control animals died after the intraperitoneal injection.

As the content and proportion of gut proteases differ among organs and species, the ability of PmPV2 to withstand proteolytic activity *in vivo* was standardized using BSA as a control. In agreement with the *in vitro* resistance of PmPV2 to proteases, after oral administration at 2 and 6 h, higher amounts of PmPV2 were detected in the small intestine (2 h: *P* < 0.0001 6 h: *P* = 0.0291 vs. BSA-administered mice). Moreover, at 2 and 6 h the ratio of PmPV2:BSA was 2.84 and 4.95, respectively, while at 8 h, only a slight difference in the amount of PmPV2 vs. BSA was observed (Figure 5A).

To understand the cytotoxic effect and the mechanism of entry of PmPV2, we studied whether internalization was occurring on inductive sites of the intestinal mucosa, the Peyer's patches (PPs). Interestingly, 2 h after inoculation a high amount of PmPV2 was detected in PPs (*P* = 0.027 vs. BSA administered mice), while at 6 h a non-significant increase was observed. As in the small intestine experiment, the PmPV2:BSA ratio was 3.6 and 7.8 at 2 and 6 h after administration, respectively, indicating that at these time periods a significant amount of PmPV2 was internalized in PPs (Figure 5B). In concordance with this latter result, PmPV2 effects on PPs *in vivo* were observed. Whereas, control animals showed regular, non-reactive lymphoid follicles (Figure 5C), treated animals had lymphoid follicles with pallid germinal center indicating induction of immune reaction (Figure 5D). In these secondary lymphoid follicles, cumuli of apoptotic bodies reactive to caspase-3 antibodies were observed (Figure 5D,*inset*), indicating that PmPV2 could also be toxic through apoptosis in some cell populations.

### Oral Immunization

In agreement with PmPV2 internalization and detection in lymphoid follicles of the PPs, anti-PmPV2 IgG was detected in the sera of mice gavaged with 0.8 mg of PmPV2 (Figure 5E). Further, all the immunized mice survived to an i.p. injection of 1 mg of PmPV2 which is about 200% of its reported LD50 without any signs of intoxication (Figure 5F). Control groups showed no reaction to IgG detection and, when injected with PmPV2, all of the neurological signs previously reported were observed and all mice died after 48 h.

## Discussion

Eggs are usually an unattended life-cycle stage in gastropods and depend entirely on the defensive compounds maternally transferred that ensure embryos normal development and protection against pathogens. Notably, two distinctive immune-related polypeptides were found in the eggs of two *Pomacea* species, *P. canaliculata* and *P. maculata*: a tachylectin (PV2-31) and a MACPF-containing protein (PV2-67) (4, 5), which are combined into the perivitellin PV2 complex (8, 9). Comparative genomic analysis together with expression patterns and proteomic validation showed that although these lectin and MACPF are present in the genomes of four species of the family, as well as in the genomes of other Mollusks, only in *Pomacea* these two proteins experienced extensive gene expansion by tandem duplication and neofunctionalization into the PV2 complex, which is expressed as such only in an accessory gland of females and transferred to eggs (30). Although the immune role of these two proteins are largely unexplored in snails, a PV2-67-like protein found in the kidney of the snail *Littorina littorea* showed overexpression when infected with a trematode parasite (31), indicating a putative immune function in the common ancestor of mollusks MACPF (12). In addition to their immune role, another prominent role of animal MACPFs is in the embryonic development of several organisms, ranging from sea urchins to mammals (32). Similar to those proteins PmPV2 is maternally transferred to the eggs, where it is massively accumulated during the early developing stages, before the embryo consumes it (33). However, PV2 structure lacks some key structural features described in developmental MACPFs, such as absence of ancillary domains and shorter TMH1 (12, 32). Finally, a less-extended group of animal MACPFs also act as toxins such as those from some cnidarians and the stone fish, where they play a role in prey capture (22, 34). The co-option of PV2 into *Pomacea* eggs and its neurotoxicity to mice locates it within the group of MACPF toxins, although here it plays a defensive role against terrestrial predators (30). The novelty found in this work is that PV2 also exerts enterotoxic effects, a role never ascribed to MACPFs.

These *Pomacea* eggs have also other biochemical defenses targeting the digestive system, notably perivitellins that lower the nutritional value (i.e., antinutritive or indigestible) and others with antidigestive properties (i.e., digestive enzyme inhibitors) (9, 23, 27, 28, 35, 36). These noxious proteins, advertised by a warning (aposematic) pink-reddish coloration, seems to be an effective passive defense system since eggs have virtually no predators, except for the fire ant, *Solenopsis geminata* (37). Here we report a novel enterotoxic role for PV2, previously described as a neurotoxin (8).

Akin to other perivitellins, we found that PmPV2 is highly stable at pH values ranging from 4.0 to 10.0, a range that includes most digestive system environments of animals (38, 39). The increases in *Rg* and *Dmax* without changes in mass or secondary structure observed at pH 4.0 may be explained as a partial quaternary unfolding of some protein domains; a behavior already documented on model proteins like BSA using SAXS (40). In this regard, several reports in other pore-forming proteins (PFPs) suggest that the acidic microenvironment found at the membrane vicinity partially denatures the pore-forming domain to a more flexible state, leading to the conformational changes needed for membrane insertion (41–45). Moreover, it is worth to mention that the small intestine of mice has an acidic pH (<5.2) relatively close to this experimental condition (46). However, whether these structural changes associated with pH are related with the PmPV2 function needs to be confirmed. We also demonstrate that it is resistant to the proteolytic activity of common digestive enzymes *in vitro*, and to gastrointestinal tract enzymes *in vivo* (see below), indicating that it is refractive to digestion and assimilation by predators. The non-digestible property of PV2, not only contributes to lower the nutritional value of eggs for a predator, but also allows PV2 to reach its intestinal tract in an active form to exert its toxic effect. The effects of the purified toxin on the gut are similar to those reported for diets supplemented with *P. canaliculata* PVF (23, 24), indicating that the PV2s are responsible, at least of some of the reported gut alterations. After reaching the intestinal lumen, oral toxins must either traverse the intestinal barrier to reach their target cells or exert its toxicity on the gut. In this regard, we provide evidence that PmPV2 does both. First it binds to intestinal cells and then induces strong morphological changes and cell detachment in a similar way as bacteria Cholera toxins (CT), Shiga toxins (Stxs) and heat-labile (LT) enterotoxins do (47). Then PmPV2 has a cytotoxic effect triggered by its structural components, because, like the above-mentioned bacterial toxins (10, 11, 13), PV2s have an AB-toxin structure, with a ¨B¨ lectin unit that delivers a toxic MACPF ¨A¨ module to the target cell (9, 12). Among the morphological changes that PmPV2 causes on Caco-2 cells, a notorious increase in plasma membrane roughness was observed by AFM. According to the bibliography, this increase could be due to three main reasons: (1) the formation of holes produced by the insertion of protein molecules into the membrane, as was observed for this and other PFPs (12, 48); (2) membrane vesiculation during protein internalization, a process commonly observed in AB toxins (10, 11, 49); (3) membrane and cytoskeleton reorganization as usually displayed by host cells in response to membrane damage (50–52). Further analyses are needed to confirm which of these processes -alone or combined- are triggered by PV2 toxins.

The ability of PmPV2 to bind to and kill enterocytes suggested a putative enterotoxic role for this toxin. Cytotoxic enterotoxins kill target cells through either lytic or non-lytic mechanisms by inducing necrosis or apoptosis, respectively (13, 47). Here, we demonstrate that PmPV2 triggers necrosis in Caco-2 cells, the same cell death pathway reported for bacterial pore-forming toxins such as alpha hemolysin (HlyA), staphylococcal alpha-toxin, pneumolysin, streptolysin-O and leukotoxin PFTs (15, 53). Interestingly, cells treated with PmPV2 showed two subpopulations of necrotic cells, which can be interpreted as different stages of cell damage. It has been reported that permeated living cells -which become transiently defective before total loss of the ability to exclude the dye- showed moderate labeling in comparison to the intensive labeling of dead cells (54). Besides necrosis, when using a rodent as a predator model, apoptotic bodies were also observed in lymphoid follicles after incorporation of the toxin indicating that another toxic mechanism is also present, an observation that requires future research to clarify.

In the present work, we were also able to detect orally ingested PmPV2 attached to the enterocyte glycocalyx of small intestine, indicating that the toxin reaches the intestinal mucosa in an active form. Binding to the mucosal surface may further protect this egg toxin from luminal digestive proteases as reported for plant enterotoxins (19). After binding, PmPV2 induced strong morphophysiological changes in the small intestine mucosa in <24 h. Several of these effects resemble those caused by plant seed dietary lectins, where toxicity is mainly attributed to interference with the digestive process and to anatomic abnormalities after binding to cell surface glycans on enterocytes (55, 56). We also demonstrate that PmPV2 not only causes morphological changes but also increases the permeability of enterocyte monolayers, indicating that it is able to disrupt the intestinal barrier. This provides an explanation of previous reports showing that oral administration of *P. canaliculata* PVF induced an increase of total absorption rate affecting both paracellular (i.e., between enterocytes) and transcellular (i.e., through enterocytes) pathways (24). This increased intestinal permeability may generate an uncontrolled income of dietary macromolecules (57, 58) further contributing to PV2 toxicity. Besides, the presence of high amounts of PmPV2 in the Peyer's patches suggests that it may also be entering the predator's body through a different pathway. Results suggest it may traverse the barrier through M cells, cells involved in the modulation of mucosal and systemic immune responses (59). In fact, lymphoid follicle activation was observed in treated mice. Independently of the mechanism through which PmPV2 traverses the intestinal barrier, ingestion of minute amounts (~0.8 mg) stimulated the immune system of mice. This small amount of PV2 is biologically relevant if we consider that a single egg-clutch contains ~67 mg PV2 (26). The immune response generated by PmPV2 was characterized by the presence of specific IgG antibodies that protected mice from an otherwise lethal injection of PV2, and also by the presence of apoptotic immune cells in the Peyer's patches. Remarkably, a similar response has been described for both invasive bacterial enterotoxins and plant dietary lectins ingested by mice and rats (14, 15, 19, 60, 61), pointing to a convergent mechanism among plant, bacteria, and animals' proteinaceous toxins. The resemblance between *Pomacea* eggs and plant seed defenses could be understood from an ecological point of view: both seeds and eggs are usually unattended life stages that are often exposed to similar selective pressures by predators and pathogens.

As a whole, these results indicate that PmPV2 not only affects the nervous system but also targets the digestive system. To our knowledge, there is no report of animal toxins with such dual effect. Furthermore, toxins having enterotoxic and neurotoxic activities at the same time were only reported in one bacterium, Stxs from *Shigella dysenteriae* (62). However, it is notable that the oral administration of PmPV2 did not cause the neurological signs observed when it is intraperitoneally injected. This absence of neurotoxicity through oral administration could be due to many causes still unclear.

Nowadays, there is an increasing biomedical interest on molecules capable of withstanding the harsh gastrointestinal environment and inducing immune responses to be used as adjuvants for oral vaccination (61, 63). Currently, bacterial enterotoxins (like CT from *Vibrio cholerae* and LT from *Escherichia coli*) or their attenuated derivatives are mostly used for this purpose (64–66). The results gathered here indicate that PmPV2 is a good candidate to be tested as an adjuvant for oral vaccine design.

It is interesting to recall that other species of the *Pomacea* genus that lack PV2 enterotoxin have evolved different protective perivitellins such as the lectin PsSC from *P. scalaris* whose ingestion affects gut morphology but in a non-cytotoxic way (36). This suggests that it is likely that there are still other enterotoxic compounds yet to be discovered within the well-protected eggs of this rapidly diversifying group.

## Conclusion

Avoiding attack is essential for survival and, under this selective pressure organisms have evolved a plethora of mechanisms to deter predators. In this study we unveiled part of the multiple defenses of *Pomacea* snails that suggest that the cooption of new functions in immune related egg proteins confer an advantage for survival and, even, diversification and spread of this highly invasive species.

By combining a lectin and a pore-forming protein, *Pomacea* PV2s have acquired enterotoxic properties, a role that has never been ascribed to lectins or perforin protein families in animals. This is also the first example of a eukaryotic toxin having both neuro- and enterotoxic activities. Finally, this work provides the first description of a true animal enterotoxin, extending by convergent evolution the presence of plant- and bacteria-like enterotoxins to unattended reproductive stages in animals and expanding the varied roles of MACPF in nature.

## Methods

### Purification and Fluorescent Labeling of PmPV2

PmPV2 was purified from newly laid *P. maculata* egg clutches as previously described (8). Total protein was quantified following the method of Lowry et al. (67) using a standard curve prepared with bovine serum albumin (BSA) (Sigma-Aldrich, St. Louis, MO, USA). The protein was labeled using the Alexa Fluor 488 Protein Labeling Kit (Life Technologies-Molecular Probes, Eugine, OR, USA) following manufacturer instructions. Labeled BSA (Life Technologies-Molecular Probes, Eugine, OR, USA) was employed as negative control.

### Cell Culture

For experimental analysis, we used the Caco-2 line of human colorectal adenocarcinoma cells, commonly used as a model of intestinal physiology and toxicology (68). Caco-2 cells were cultured in Dulbecco's modified Eagle's medium (DMEM) with 0.45% (w/v) D-glucose and supplemented with 10% (v/v) newborn calf serum, penicillin/streptomycin, amino acids and vitamins (Life Technologies-Invitrogen, Gaithersburg, MD, USA). Cells were cultured at 37°C in a humidified atmosphere of 5% CO_2_. The culture medium was replaced every 2 days. After reaching 95% confluence, cells were subcultured by trypsinization. Cell viability was checked by trypan blue exclusion assay (69). Passages from 80 to 105 were used, with a window no higher than 10 passages within each experiment. All experiments were conducted with a confluence of cells above 90%.

### Mice

BALB/c AnN mice, *Mus musculus* Linnaeus, 1758 (body mas = 20.2 ± 1.7 g), were obtained from the Experimental Animals Laboratory of the School of Veterinary Science, UNLP. All experiments were performed in accordance with the Guide for the Care and Use of Laboratory Animals (70) and were approved by the Comité Institucional de Cuidado y Uso de Animales de Experimentación (CICUAL) of the School of Medicine, UNLP (Assurance No. P08-01-2013).

### PmPV2 Resistance to *in silico* and *in vitro* Gastrointestinal Digestion

Before the *in vitro* assay, we analyzed the protein digestibility *in silico* using the amino acid sequences of both PmPV2 subunits already published (5). The number of putative cleavage sites of pepsin and trypsin was determined using the PeptideCutter server (https://web.expasy.org/peptide_cutter/) (71).

#### Gastric Phase

A simulated gastrointestinal digestion of PmPV2 was performed using the method described by Moreno et al. (72) with some modifications (9, 27). Briefly, PmPV2 in Mili-Q water was dissolved in simulated gastric fluid (0.15 M NaCl, pH 2.5) to a final concentration of 0.5 μg/μL. The gastric phase was conducted at 37°C in the presence of porcine pepsin (Sigma-Aldrich) at an enzyme:substrate ratio of 1:20 (w/w). Aliquots of 5 μg protein were taken at 0, 60 and 120 min after the addition of the pepsin. The reaction was stopped by increasing the pH with 150 mM Tris-HCl buffer pH 8.5.

#### Intestinal Phase

For *in vitro* duodenal digestion, 100 μL of the 120 min gastric digest was used as starting material. The pH of the digests was adjusted to 8.5 with 0.1 M NaOH and the following were added: 22.8 μL of 0.15 M Tris/HCl buffer (pH 8.5) and 4.17 μL of 0.25 M sodium taurocholate (Sigma-Aldrich) solution. The simulated duodenal digestion was conducted at 37°C using bovine pancreas trypsin (Sigma-Aldrich) at an enzyme:substrate ratio of 1:2.8 (w/w). Aliquots were taken at 0, 60, and 120 min.

#### Electrophoretic Analysis

Samples taken from both gastric and duodenal phases were immediately boiled for 10 min in SDS electrophoresis buffer with β-mercaptoethanol (4%) and analyzed by SDS-PAGE using 4–20% gradient gels prepared following the Laemmli (73) method. Gels were stained with Coomassie Brilliant Blue G-250 (Sigma-Aldrich). BSA (Sigma-Aldrich) in the presence and in absence of enzymes was used as positive and negative controls, respectively.

### Structural Stability Against pH

Structural stability against pH was determined in PmPV2 (65 μg/mL) at pH values ranging from 2.0 to 12.0. Buffers of the desired pH were prepared using sodium phosphate salts and citric acid buffers (74). After *overnight* incubation, samples were analyzed by fluorescence spectroscopy, CD, and SAXS as follows.

#### Fluorescence

Fluorescence emission spectra of PmPV2 (65 μg/mL) in PBS buffer (1.5 mM NaH_2_PO_4_, 8.1 mM Na_2_HPO_4_, 140 mM NaCl, 2.7 mM KCl, pH 7,4) were recorded in scanning mode in a Perkin-Elmer LS55 spectrofluorometer (Norwalk). Protein was exited at 280 nm (4 nm slit) and emission recorded between 275 and 437 nm. Fluorescence measurements were performed in 10 mm optical-path-length quartz-cells. The temperature was controlled at 25 ± 1°C using a circulating-water bath.

#### Circular Dichroism

Spectra of PmPV2 (70–140 μM) were recorded on a Jasco J-810 spectropolarimeter using quartz cylindrical cuvettes of 1-mm or 10-mm path lengths for the far-UV (200–250 nm) and near-UV (250–310 nm) regions, respectively. Data were converted into molar ellipticity [θ]_M_ (deg.cm^2^. dmol^−1^) using a mean residue weight value of 115.5 g/mol for PmPV2.

#### Small-Angle X-Ray Scattering (SAXS)

Synchrotron SAXS data from solutions of PmPV2 at different pH values (74) were collected on the SAXS2 beamline at the Laboratório Nacional de Luz Sincrotron (Campina, Brazil) using MAR 165 CDD detector at a sample-detector distance of 1.511 m and at a wavelength of λ = 0.155 nm (I_(s)_ vs. s, where s = 4πsinθ/λ, and 2θ is the scattering angle). Protein concentrations ranging between 0.8 and 2 mg/mL were measured at 20°C; BSA (Sigma-Aldritch) was measured as a molecular mass standard. Five successive 300-s frames were collected. The data were normalized to the intensity of the transmitted beam and radially averaged; the scattering of the solvent-blank was subtracted. Radius of gyration (*Rg*), molecular mass and maximum intraparticle distance (*D*_*max*_) were estimated from the final curves using ATSAS 3.0.1 (r12314) (75). Molecular mass was estimated from intensity at s = 0 (I_0_) of the sample and reference (BSA) calculated by the software.

### PmPV2 Interaction With Caco-2 Cells

#### Binding Assay

Caco-2 cells were seeded on a 24-well plate (Greiner Bio-One, Monroe, NC, USA) and were incubated at 37°C for 48 h. Then, cells were washed twice with PBS (1.5 mM NaH_2_PO_4_, 8.1 mM Na_2_HPO_4_, 140 mM NaCl, 2.7 mM KCl pH 7.4) and incubated with Alexa488-labeled PmPV2 or BSA in PBS (0.4 mg/mL) for 1 h at 37°C. Cells were observed in an inverted fluorescence microscope (Olympus IX-71).

#### Effect of PmPV2 on Cell Morphology

Changes in Caco-2 cell morphology and membrane roughness was determined by atomic force microscopy (AFM) following the protocol of Cattaneo et al. (76). Cells were cultured on slide covers and incubated at 37°C for 24 h. Then, the medium was replaced by a PmPV2 solution in DMEM (0.05 μg/μL) and incubated at 37°C for 24 h. After treatment cells were washed twice with PBS and fixed using an ethanol dehydration train (35°, 45°, 55°, 75°, 85°, 96°, and 100°) at room temperature and air-dried (77). Cells were photographed before and after fixation to check any morphological effect due to this step. Six different cell samples (three controls and three treated) were analyzed by AFM in air, using a MultiMode Scanning Probe Microscope (Veeco Instruments Inc., Santa Barbara, CA, USA) coupled with a Nanoscope V controller (Veeco Instruments Inc.). Measurements were obtained with Tapping® mode, using probes doped with silicon nitride (RTESP, Veeco Instruments Inc., with tip nominal radius of 8–12 nm, 271–311 kHz, force constant 20–80 N/m). The typical scan rate was 0.5 Hz. The analysis was performed on either a large area (50 × 50 μm) or a smaller area of the cell surface of 15 × 15 μm^2^ and 5 × 5 μm^2^. Membrane roughness was evaluated in 5 × 5 μm^2^ sections taking into consideration the *Ra, Rq* and *Image Surface Area Difference (ISAD)* parameters determined using the Nanoscope Analysis 1.5 software package. The parameter *Ra* is the arithmetic mean of the deviations in height from the roughness mean value, *Rq* is the root mean square of the height distribution, and *ISAD* is the difference between the tridimensional area and the bidimensional area.

#### Cytotoxicity

The cytotoxic effect of the PmPV2 on enterocytes was evaluated on Caco-2 human colorectal adenocarcinoma cells following the method described in Dreon et al. (23). In brief, once cell cultures reached the desired confluence, 50 μl/well of a 2-fold serial dilution of PmPV2 (0.111 mg/mL) in PBS were added and incubated at 37°C for 24 h. Control wells were prepared with 50 μL/well of PBS. Cell viability was measured using the 3-(4,5-dimethythiazol-2-yl)-2,5-diphenyl tetrazolium bromide (MTT) assay (78), in a microplate Multimode Detector DTX-880 (Beckman Coulter, Inc., CA, USA). Cell viability was expressed as control percentage: % Viability = (OD treated cells/OD control cells) × 100.

#### Apoptosis vs. Necrosis Assay

Knowing that the PmPV2 was cytotoxic to Caco-2 cells we performed an assay to determine whether the toxin induces apoptosis or necrosis. Caco-2 cells were cultured in 6-well plates (Greiner Bio-One) and incubated at 37°C for 24 h. Then 100 μL of a PBS solution containing 100 μg of PmPV2 was added 24, 12, 3, 1, 0.5, and 0.25 h before trypsinization; 100 μL of PBS buffer was used as control. After harvest, cell suspensions were washed two times and resuspended in binding buffer (10 mM HEPES, 140 mM NaCl, 2.5 mM CaCl_2_ pH 7.4) at 2 × 10^6^ cells/mL. Next, 5 μL of annexin V-FICT (BD Biosciences, San Jose, CA, USA) and 10 μL of propidium iodide (PI, BD Biosciences) solutions were added to 100 μL of each cell suspension and incubated at room temperature for 15 min in the dark. After adding 300 μL of binding buffer and stirring, cell fluorescence was determined immediately using a BD FACSCalibur flow cytometer (BD Biosciences) and data were analyzed with Flowing Software v. 2.5.1. The percentage of apoptosis was taken as the percentage of only annexin V-positive (FICT^+^/PI^−^), the percentage of necrosis was either double-positive (FICT^+^/PI^+^) or only PI-positive cells (FICT^−^/PI^+^), while percentage of viable cells was double-negative cells (FICT^−^/PI^−^).

#### Transepithelial Electric Resistance (TEER) Assay

A volume of 500 μL of a Caco-2 cell suspension was cultured in 8.4 mm ThinCert transwell system of 0.4-μm pore size (Greiner Bio-One) for 24-well plates. Two milliliters of medium were added to each well. The medium was changed every 2 days in both compartments. Changes in TEER were followed by measuring the culture every 24 h using an EVOM-Epithelial voltohmmeter (World Precision Instruments Inc., Sarasota, FL, USA) until resistance reached a constant value (i.e., the monolayer was completely formed). Then, 100 μL of a 10-fold serial dilution of PmPV2 (2 mg/mL) in PBS was added. TEER was measured at different times within 96 h post-treatment. Data were expressed as percentage of TEER relative to the starting value at 0 h.

### PmPV2 Interaction With Mice Intestine

#### Histological Analysis

Small intestine morphology was analyzed in three groups of treated mice, six animals each (three males and three females). One group was gavaged with a single dose of 300 μL of PBS containing 400 μg (<1% of the amount found in one clutch) of PmPV2 (1-dose group). Another group of mice was gavaged with doses of 300 μL of the same solution every 24 h during 4 days before intestine extraction (4-dose group). The control group (6 mice) received the equivalent volume of PBS. Oral gavage was performed using a winged needle infusion set and was completed within 30 s. Twelve hours after the single or last dose the intestines of the mice were removed and cylindrical tissue samples were fixed and analyzed as previously reported (24). Samples were stained using hematoxylin-eosin or PAS. PmPV2 binding to intestinal mucosae was analyzed by immunohistochemistry (IHC) using rabbit IgG anti-PcPV2 antibodies following previous reports (9). Apoptosis was also detected by IHC using mouse IgG anti-Caspase 3 monoclonal antibodies diluted 1:100 in PBS buffer (Santa Cruz Biotechnologies Inc., Santa Cruz, CA, USA).

#### Fate and Internalization of PmPV2 *in vivo*

To evaluate PmPV2 fate and internalization Alexa488-labeled PmPV2 and BSA in PBS were used. Three sets of nine mice (females and males) each were gavaged with (*i*) PBS, (*ii*) Alexa488-labeled BSA (75 μg) as control, or (*iii*) Alexa488-labeled PmPV2 (75 μg). At 2, 6 and 8 h post-administration three mice of each treatment were sacrificed and Peyer's patches (PPs) (9 PPs/mice) and the remaining small intestine (SI) were aseptically removed. SI was homogenized with 5 mL of PBS in a Potter type homogenizer OS-40 Pro (DLAB Scientific Inc., Riverside, CA, USA). Single-cell suspensions were prepared (79) from PPs and washed twice in PBS solution in order to remove extracellular labeled protein. Cell suspensions were resuspended in 5 mL of PBS. Then 100 μL of homogenate or cell suspensions were seeded in 96-well, flat-bottom, black microplate (Corning Inc., Corning, NY, USA) and fluorescence was measured in a microplate reader Multimode Detector DTX-880 (Beckman Coulter Inc., Brea, CA, USA). Arbitrary fluorescence units (A.U.) were corrected by labeling efficiency (effi) and molarity (A.U./effi.mol).

### Oral Immunization

Groups of three female mice each were gavaged with two boosters of 100 μL of PBS containing 800 μg of PmPV2 with 19 days between each other; mice gavaged with 100 μL of PBS were used as control (*n* = 3). Immunization was determined by measuring IgG anti-PmPV2 in serum and by analyzing mice resistance to PmPV2 lethal toxic effect. Three days after the second boost blood was sampled by cheek puncture and serum obtained as previously described (9) and kept at −70°C until used. Spots of 0.05 μg of PmPV2 were pipetted onto nitrocellulose membranes (GE Healthcare-Amersham Biosciences Inc., Piscataway, NJ, USA) and membranes blocked with 5% (w/v) non-fat milk in PBS with 0.05% (v/v) Tween 20 (Anedra S.A., San Fernando, BA, Argentina) (PBST) at 4°C overnight. Then membranes were incubated with anti-sera solutions (1:100) in 3% (w/v) non-fat milk in PBST. After washing 5 times with PBST for 5 min each time, the presence of anti-PmPV2 antibodies was detected using anti-mouse IgG horseradish peroxidase conjugate (1:3,000, Bio-Rad Laboratories Inc., Hercules, CA, USA). Membranes were washed as above and revealed by chemiluminescence. Twelve days after the second boost, mice were intraperitoneally (i.p.) injected with 200 μL of a PBS solution containing 22.4 μg of PmPV2 (i.e., 1 mg/kg), a dose 4 times higher than the reported LD50, 96 h (8). Animals were observed every day for 4 days to check for neurological signs and survival.

### Statistical Analysis

Statistical analyses were conducted with GraphPad PRISM v. 5.03 software and results expressed as mean ± 1 SEM. Mucosal-to-serosal amplification ratio (*M*), TEER and absorption parameters were determined by one-way analysis of variance (one-way ANOVA) with *post-hoc* Bonferroni's test. The significance level selected to accept difference for all statistical analysis performed was α < 0.05.

## Data Availability Statement

All datasets generated for this study are included in the article/Supplementary Material.

## Ethics Statement

The animal study was reviewed and approved by Comité Institucional de Cuidado y Uso de Animales de Experimentación (CICUAL) of the School of Medicine, National University of La Plata (Assurance No. P08-01-2013).

## Author Contributions

AI, HH, SI, and MG designed the research. AI, PF, SI, MG, EP, and MD performed research. AI, PF, SI, EP, MD, MG, and HH analyzed data and revised the draft. AI, MG, and HH drafted the article.

### Conflict of Interest

The authors declare that the research was conducted in the absence of any commercial or financial relationships that could be construed as a potential conflict of interest.

## Abbreviations

MACPF: Membrane Attack Complex/Perforin
PFT: pore-forming toxin
PVF: perivitelline fluid
PV2: perivitellin-2
SAXS: small angle X-ray scattering
CD: circular dichroism
BSA: bovine serum albumin
ISAD: image surface area difference
TEER: transepithelial electric resistance
PPs: Peyer's patches
i.p: intraperitoneally
CT: cholera toxin
Stxs: Shiga toxins
LT: heat-labile enterotoxin
HlyA: alpha-hemolysin
PsSC: scalarin.
