# Supplementary material for: Novel Role for Animal Innate Immune Molecules: Enterotoxic Activity of a Snail Egg MACPF-Toxin
Source: Front Immunol. 2020 Mar 13;11:428. doi: 10.3389/fimmu.2020.00428 (PMC7082926; doi:10.3389/fimmu.2020.00428)
Supplement: Supplementary file 1 [file Table_1.DOCX]

Supplementary Material

# Supplementary Figure


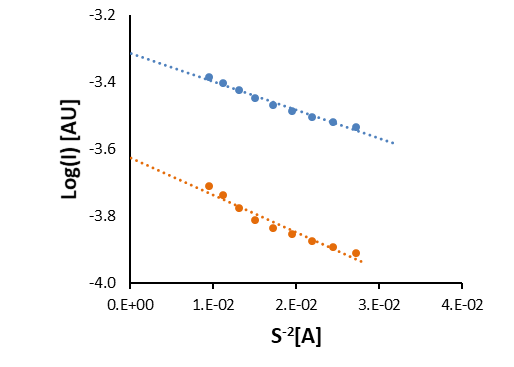


MM= 115 kDa

MM= 143 kDa


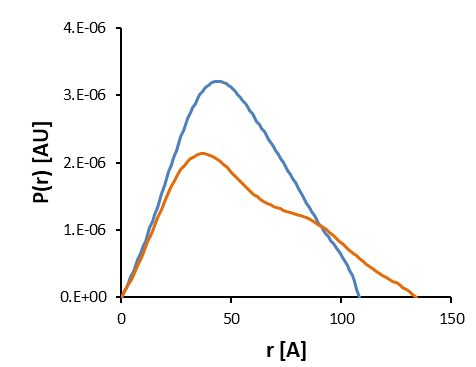


**A**

**B**

**Figure S1. SAXS analysis of PmPV2 *Dmax* and molecular mass at different pH values**. **A:** Pair distance distribution of PmPV2 showing the increase (arrow) in the maximum intramolecular distance (*Dmax*) – i.e. maximum r value. **B:** Molecular mass (MM) estimation of PmPV2 by the S=0 (I_0_) method using BSA as reference. Samples were analyzed using ATSAS 3.0.1 (r12314) software. Blue: PmPV2 at pH 6.0; Orange: PmPV2 at pH 4.0.
